# Insulin for type 2 diabetes: choosing a second-line insulin regimen

**DOI:** 10.1111/j.1742-1241.2008.01909.x

**Published:** 2008-11

**Authors:** A Barnett, A Begg, P Dyson, M Feher, S Hamilton, N Munro

**Affiliations:** 1 University of Birmingham and Heart of England NHS Foundation TrustBirmingham, UK; 2 Links Health CentreMontrose, UK; 3 Oxford Centre for Diabetes, Endocrinology and MetabolismOxford, UK; 4 Beta Cell Diabetes Centre, Chelsea and Westminster HospitalLondon, UK; 5 Queen Mary’s HospitalRoehampton, London, UK; 6 Little Orchard Surgery, Leatherhead and Chelsea and Westminster HospitalLondon, UK

## Abstract

Guidance has been published on the choice of initial insulin regimen for patients with type 2 diabetes [NPH (isophane) insulin or a long-acting insulin analogue] but not on how to choose a second regimen when glycaemic control becomes unsatisfactory.

**Aims:**

To develop pragmatic clinical guidance for choosing a second-line insulin regimen tailored to the individual needs of patients with type 2 diabetes after failure of first-line insulin therapy.

**Methods:**

Formulation of a consensus by expert panel based on published evidence and best clinical practice, taking into account patient preferences, lifestyle and functional capacity.

**Results:**

Six patient-dependent factors relevant to the choice of second-line insulin regimen and three alternative insulin regimens (twice-daily premixed, basal-plus and basal-bolus) were identified. The panel recommended one or more insulin regimens compatible with each factor, emphasising the fundamental importance of a healthy lifestyle that includes exercise and weight reduction. These recommendations were incorporated into an algorithm to provide pragmatic guidance for clinicians.

**Conclusion:**

The three alternative insulin regimens offer different benefits and drawbacks and it is important to make the right choice to optimise outcomes for patients.

**What's known**

- Treatment of type 2 diabetes aims to maintain glycaemic control as beta cell function declines by escalating drug treatment from monotherapy (usually with metformin) to combined treatment (usually with a sulfonylurea).
- Many patients ultimately require insulin. NICE recommends NPH (isophane) insulin as the insulin of first choice, although in practice many clinicians prescribe a long-acting insulin analogue.
- There is no guidance on choosing a second-line regimen when initial therapy fails.

**What's new**

- There are three alternative regimens for second-line insulin therapy: twice-daily premixed; basal-bolus (once-daily injection of a long-acting insulin plus injections of a short-acting preparation at every meal) and basal-plus (basal insulin plus one or two meal-time injections).
- The choice of regimen should be tailored to patient need, as reflected by six factors (preference for injection frequency and self-monitoring blood glucose, variability of lifestyle, presence of postprandial hyperglycaemia, patient’s capability and access to support).
- An algorithm has been developed to help clinicians choose an appropriate insulin regimen.

Type 2 diabetes is a progressive disorder associated with declining pancreatic beta cell function and increasing insulin resistance. This often results in the need for combination therapies in order to maintain target HbA_1C_ by escalating drug treatment from monotherapy (usually with metformin) to combination therapies on a platform of healthy lifestyle and weight control.

All patients should receive education about their disorder and be encouraged to adopt a healthy lifestyle and lose excess weight but, despite the continuing need for a healthy lifestyle, most require drug treatment. The UKPDS study showed that only 25% of newly diagnosed patients could maintain target HbA_1C_ after 3 years using diet alone; this declined to 9% after 9 years (1). The aim is to maintain target HbA_1C_ as beta cell function declines by escalating drug treatment from monotherapy (usually with metformin) to combined treatment (usually with a sulfonylurea).

From the recent NICE guidance, if glycaemic control remains inadequate, the next step is to add treatment with insulin, a glitazone or exenatide, the choice depending on both clinical factors and patient preference. Many patients with type 2 diabetes require insulin to maintain glycaemic control. In UK general practice, it is estimated that only half of patients who need insulin after failure of oral agents will receive it within 5 years (2). The median time from beginning treatment with the last oral agent to beginning insulin therapy is approximately 8 years (3).

The NICE guidance (4) recommends initiating insulin with NPH (isophane) insulin or a long-acting analogue to provide a basal insulin supply (basal insulin) and includes advice on the choice of initial insulin. A summary of the different types of insulin is presented in Box 1.

**Box 1:** Summary of types of insulins

Type of insulin	Summary of properties	Examples (not exhaustive)
Short-acting insulins	Rapid onset but short duration of action suitable for injectionbefore meals; analogues (insulin aspart, insulin glulisine, insulin lispro)have faster onset and shorter duration than soluble insulin	*Analogues*
		Apidra
		Humalog
		NovoRapid
		*Soluble human insulin*
		Actrapid
		Humulin S
		Insuman Rapid
Premixed insulins(biphasic insulins)	Combination of insulins with complementary durations ofaction – e.g. a short-acting (soluble insulin, insulin aspart,insulin lispro) plus an intermediate-acting insulin(aspart or lispro protamine insulin, protamine insulin)	Humalog Mix25, Mix50
		Humulin M3
		Insuman Comb 15, 25, 50
		Mixtard30
		NovoMix30
Basal and NPH insulins	Basal insulins have a prolonged duration of action for onceor twice daily injection (insulin detemir, insulin glargine,insulin zinc suspension, protamine zinc suspension)	Hypurin Bovine Lente,
		Hypurin Bovine Protamine Zinc
		Lantus
		Levemir
	NPH insulins are a complex of bovine or porcine insulin,or human insulin, with protamine; intermediate duration of action	Humulin I
		Insulatard
		Insuman basal

NPH, isophane insulin.

Glycaemic control with the initial insulin regimen is suboptimal for the majority of patients: 6 months after starting insulin, HbA_1C_ is still 7.5% or higher in 74% of patients (2) and after 1 year below 6.5% in 24% or fewer (5). NICE states that, if target HbA_1C_ with the initial regimen is not reached without problematic hypoglycaemia, patients using a basal regimen should consider additional meal-time doses or switching to a premixed insulin. For those already using a premixed insulin once or twice daily, it suggests they should consider an additional meal-time injection or change to a basal regimen plus meal-time injections. A regimen comprising once-daily basal long-acting insulin plus meal-time injections of a short-acting insulin is known as a basal-bolus regimen. The panel used the term ‘basal-plus’ to describe a regimen comprising a once-daily basal insulin plus one or two meal-time injections of a short-acting insulin.

There is currently no guidance on how to implement this step in insulin treatment in a way that is tailored to the needs of individual patients. This reflects a lack of randomised trials from which to develop an evidence-based strategy. In May 2008, an expert panel (comprising the authors) met to review current clinical practice with the aim of developing pragmatic advice that is easy to use during a consultation and will help general practitioners (GPs) tailor treatment to the needs of individual patients.

## Target HbA_1C_

Good glycaemic control reduces the risk of cardiovascular and microvascular complications but HbA_1C_ levels within the normal range may be achieved only with very intensive treatment that carries an unacceptable risk of adverse effects for many people. Among patients who achieve a target HbA_1C_ of ≤ 6.5%, almost half using biphasic insulin and over one-fifth of those using basal insulin experience hypoglycaemic events that are symptomatic or require assistance (5). There is concern that treatment with basal insulin plus a sulfonylurea may also be associated with an increased risk of hypoglycaemia.

Patients should therefore be involved in deciding which HbA_1C_ level is the most appropriate target for them. Although a target of 6.5% is generally recommended, the benefits of a low HbA_1C_ must be balanced against the risk of hypoglycaemia and weight gain and the potential impact of treatment on quality of life. Highly intensive management to achieve a target below 6.5% should be avoided but any movement towards this target is beneficial (4).

The Quality Outcomes Framework (6) specifies an HbA_1C_ target of between 6.5% and 7.5%. The threshold for the purposes of auditing performance is 7.4% or less; this is not optimal clinical practice.

The Expert Panel recommends a target HbA_1C_ of < 7% if this can be achieved in clinical practice, with a further reduction to 6.5–6.9% if this can be done safely particularly from the point of view of hypoglycaemia.

## Relationship of fasting and prandial hyperglycaemia to HbA_1C_

**Figure 1:**
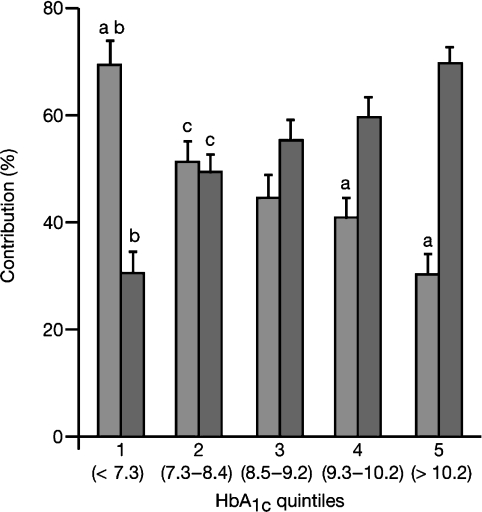
Relative contributions of postprandial and basal hyperglycaemia to overall diurnal hyperglycaemia at different quintiles of HbA_1C_ (7). 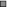, postprandial hyperglycaemia; 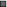, fasting hyperglycaemia; a, significant difference, fasting vs. postprandial; b, significant difference from all other quintiles; c, significant difference from quintile 5 “Copyright © 2003 American Diabetes Association From Diabetes Care®, Vol. 26, 2003; 881–885 Reprinted with permission from *The American Diabetes Association*.”

Fasting and postprandial blood glucose levels both contribute to the hyperglycaemia burden in patients with diabetes but their relative importance depends on the degree of glycaemic control (7). When control is good (HbA_1C_ < 7.3%), postprandial hyperglycaemia accounts for about 70% of overall diurnal hyperglycaemia. By contrast, when control is poor (HbA_1C_ > 10.2%), it is basal hyperglycaemia that accounts for 70% of the total (Figure 1) (7).

A once-daily long-acting basal insulin controls basal hyperglycaemia more effectively than meal-time injections of short-acting insulin; conversely, it controls postprandial glucose levels less effectively (8). As initial therapy with a basal insulin reduces HbA_1C_ towards target, this gain is likely to owe more to improved fasting glucose levels than a reduction of postprandial hyperglycaemia. While it should be acknowledged that everyone is different with respect to the balance of basal and postprandial hyperglycaemia, further movement towards target HbA_1C_ may subsequently be best achieved by adding meal-time insulin injections.

## Weight gain

Some patients may refuse insulin because of fear of or actual weight gain. Successful glycaemic control with insulin is associated with weight gain, although some patients gain more weight than others and it is less marked with once-daily basal than twice-daily or prandial insulin regimens (5). The risk of weight gain is not equal for all basal insulins; for example, once-daily insulin detemir is associated with less weight gain than insulin glargine (9). Intensive lifestyle intervention during the first 6–12 months of insulin therapy can prevent weight gain (10). Treatments other than insulin (e.g. exenatide) should be considered for patients who are obese or who have experienced marked weight gain with the initial insulin regimen (4). Some patients who need to continue insulin may require drug treatment to assist weight loss. Discontinuation of a sulfonylurea should also be considered in patients with marked weight gain. Patient education should continue to emphasise the importance of controlling weight by adopting a healthy lifestyle and diet.

## A pragmatic algorithm for choosing a second-line insulin regimen

**Figure 2:**
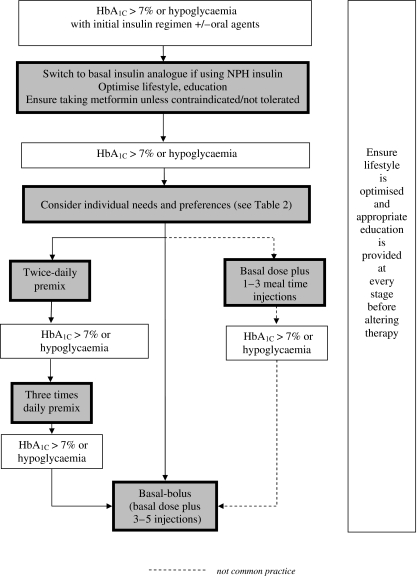
Pragmatic algorithm for choosing second-line insulin therapy in type 2 diabetes

Figure 2 is an algorithm based on a consensus of expert opinion to support decision-making when initial insulin therapy fails to achieve target HbA_1C_ or hypoglycaemic events are a significant problem. The second-line regimen should be chosen according to individual patient need.

Table 1 lists the factors believed to be most important in this decision and indicates which regimens are preferred for each.

**Table 1:** Second-line insulin choices for type 2 diabetes: patient factors

	Basal-bolus	Premix	Basal + mealtime injections
Patient preference for fewest injections		+	+
Variable meal pattern	+		+
Variable daily routine	+		
Limited capability (e.g. dexterity, cognitive function)		+	
Better postprandial glucose control required	+		+
Unwilling to self-monitor blood glucose several times daily		+	
Limited support from family and GP		+	+

+ = preferred choice; GP, general practitioner. The preferred insulin regimen for individual patients is not the one with the most ‘+’ but one which best meets specific needs.

### Optimising current therapy

Patients will normally have received lifestyle advice and education about diabetes and the use of insulin; this should include discussion of self-monitoring blood glucose. Subject to contraindications and tolerability, they may be taking metformin and/or a sulfonylurea. The dose of metformin can be limited by adverse gastrointestinal effects; a modified-release preparation may overcome this problem.

### Education

Education about healthy diet and weight loss may improve glycaemic control without the need to alter drug therapy (11) and should be reinforced every time treatment is reviewed. If necessary this should be in addition to annual structured group programmes. Education should be provided in a way that is sensitive to the individual’s needs, culture and beliefs and can be provided by telephone or in face-to-face meetings. The panel recommends a minimum of four contacts with the patient in the first 6–8 weeks after prescribing second-line insulin therapy.

### Diet and lifestyle

Dietary advice should similarly be appropriate to individual need and encourage the use of high-fibre, low-glycaemic index carbohydrates, low fat foods and control of trans and saturated fatty acids intake. Recommendations for the content and timing of meals should be individualised. Lifestyle change should include increased physical activity and weight loss initially of 5–10% for people who are overweight.

### Initial insulin regimen

NICE recommends NPH insulin as the insulin of first choice for most patients (4). However, at a similar level of glycaemic control, a long-acting insulin analogue is associated with a lower risk of hypoglycaemia than NPH insulin in patients taking metformin (12) and patients may prefer the convenience of a once-daily basal regimen. When target HbA_1C_ cannot be met without an increased risk of hypoglycaemia, or hypoglycaemic events become troublesome as the target is approached, switching to a long-acting analogue may improve the balance of benefit and risk. Patients should be warned of the possibility that hypoglycaemic episodes may increase during adjustment of insulin therapy and that this will affect their ability to drive. Information about the requirements for driving are summarised by the DVLA in *At a Glance*; copies are available at http://www.dvla.gov.uk/media/pdf/medical/aagv1.pdf.

### Options for second-line insulin regimens

When treatment, including lifestyle and oral hypoglycaemic drugs, has been optimised but glycaemic control is still unsatisfactory, the options for second-line insulin therapy are:

- Substituting a twice-daily premixed insulin, increasing to three times daily if required. When switching from a basal regimen to a twice-daily injections of premixed insulins, the initial dose should be 80% of the final basal dose; this should then be titrated to target over 2 weeks.

OR

- Basal plus meal-time injections with a short-acting insulin analogue, beginning with one additional injection per day (determined by highest postprandial reading from blood glucose monitoring), and increasing injection frequency according to need (basal-plus); meal-time doses are adjusted according to blood glucose measurement. This regimen is not common clinical practice.

OR

- A full basal-bolus regimen (a basal dose plus injections of a short-acting insulin analogue with every meal); meal-time doses are calculated according to blood glucose measurement.

The Expert Panel did not make recommendations for choosing specific insulins. Clinical experience shows that the preferences and capabilities of patients and the support available to them are the main determinants for choosing a second-line insulin regimen (Table 1). These options should be discussed with the patient, who should participate fully in the decision to agree a regimen. GPs and practice nurses should consider requesting specialist help at any stage.

### Practical issues

#### Injection frequency

Many patients dislike frequent insulin injections (13) and self-monitoring blood glucose, which many clinicians recommend for such intensive insulin therapy, is associated with increased scores of depression and lower quality of life scores (14,15). A basal-bolus regimen requires multiple daily injections (4–6/day). Premixed insulins (2 injections/day) provide superior meal-time cover compared with the basal-plus regimen (2–4 injections/day). The basal-plus regimen also offers a transition phase for patients who are ultimately likely to use basal-bolus therapy.

#### Variable meal pattern and daily routine

Insulin requirement can be more closely matched to insulin dose by administering multiple injections of a short-acting insulin in addition to a basal dose. The duration of action of premixed insulins is too long to provide such flexibility and the ratios of the component short- and long-acting insulins cannot be adjusted to meet variable demand. These formulations carry an increased risk of hypoglycaemia if meal patterns and physical activity are not consistent. Conversely, patients with a regular lifestyle may be better suited to a premixed regimen.

#### Patient’s capability

Patients who are unable to cope well with their injections, either because of the practical obstacles to handling the pen or difficulty understanding the process of monitoring and dose adjustment, should minimise the number of doses they need, reduce the need for frequent blood glucose monitoring and use a fixed-dose regimen.

#### Postprandial hyperglycaemia

Meal-time doses of a short-acting insulin are indicated if postprandial hyperglycaemia is believed to be a particular problem. The target for postprandial glucose levels is ≤ 7.8 mmol/l; patients should be encouraged to self-monitor blood glucose to help them reach this target (16).

#### Self-monitoring blood glucose

Meal-time doses in basal-plus and basal-bolus regimens are adjusted according to blood glucose measurements, meaning multiple daily finger pricks in addition to the injections themselves. Such careful tailoring of the insulin dose comes at a price for patients with type 2 diabetes, who report increased scores of depression and lower quality of life scores (13,14). A premixed regimen may then be preferred.

#### Availability of support

More education and training is needed to introduce a basal-bolus regimen than other regimens and this has implications for practice resources. Ideally, basal-bolus regimens should only be prescribed by practices that can provide adequate support and training for their staff.

Patients who can be helped to manage their injections by a carer or nurse have a greater choice of insulin regimens. However, it is important that injections should be administered at the time the patient needs them rather than to accommodate the carer’s work schedule. Given the additional workload associated with a basal-bolus regimen, there may be a need to minimise injection frequency for individuals who do not have sufficient support.

## Summary

Type 2 diabetes is a progressive disorder associated with declining pancreatic beta cell function and increasing insulin resistance. Most patients eventually require insulin to maintain glycaemic control.

Guidance has been published on the choice of the initial insulin regimen but not on how to choose a second regimen when glycaemic control becomes unsatisfactory. The three options – premixed insulins, basal-plus and basal-bolus regimens – offer different benefits and drawbacks and it is important to make the right choice to optimise outcomes for patients.

This pragmatic guidance aims to help GPs choose the second-line insulin regimen that best meets the needs of individual patients. Taking into account patient preferences, lifestyle and functional capacity, it identifies which are the most suitable alternatives. However, it recognises the importance of emphasising the benefits of a healthy lifestyle that includes exercise and weight reduction.
